# 2-(Benzene­sulfonamido)acetic acid

**DOI:** 10.1107/S1600536808035721

**Published:** 2008-11-08

**Authors:** Muhammad Nadeem Arshad, Islam Ullah Khan, Muhammad Zia-ur-Rehman

**Affiliations:** a Department of Chemistry, Government College University, Lahore 54000, Pakistan; b Applied Chemistry Research Centre, PCSIR Laboratories Complex, Lahore 54600, Pakistan

## Abstract

The title compound, C_8_H_9_NO_4_S, is of inter­est as a precursor to biologically active sulfur-containing heterocyclic cmpounds. The crystal structure displays the classical O—H⋯O inter­molecular hydrogen bonding typical for carboxylic acids forming dimers. Symmetry-related dimers are, in turn, linked through head-to-tail pairs of inter­molecular N—H⋯O inter­actions, giving rise to a zigzag chain along the *c* axis.

## Related literature

For the synthesis and biological evaluation of sulfur-containing heterocyclic compounds, see: Zia-ur-Rehman *et al.* (2005 ▶, 2006 ▶, 2007 ▶, 2008 ▶); Wen *et al.* (2005 ▶). For related structures, see: Wen *et al.* (2006 ▶); Zhang *et al.* (2006 ▶). For bond-length data, see: Allen *et al.* (1987 ▶). For background information, see: Berredjem *et al.* (2000 ▶); Esteve & Bidal (2002 ▶); La Roche & Co (1967*a*,*b*); Lee & Lee (2002 ▶); Martinez *et al.* (2000 ▶); Soledade *et al.* (2006 ▶); Xiao & Timberlake (2000 ▶). For related literature, see: Gowda *et al.* (2007*a* ▶,*b* ▶,*c* ▶); Kayser *et al.* (2004 ▶); La Roche & Co (1967 ▶); Vaichulis (1977 ▶).
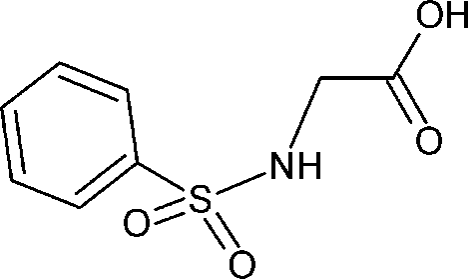


## Experimental

### 

#### Crystal data

C_8_H_9_NO_4_S
*M* *_r_* = 215.23
Monoclinic, 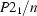

*a* = 8.5181 (3) Å
*b* = 11.1302 (4) Å
*c* = 10.6414 (4) Å
β = 112.600 (2)°
*V* = 931.42 (6) Å^3^
*Z* = 4
Mo *K*α radiation
μ = 0.34 mm^−1^
*T* = 296 (2) K
0.36 × 0.16 × 0.15 mm

#### Data collection

Bruker APEXII CCD area-detector diffractometer
Absorption correction: multi-scan (*SADABS*; Sheldrick, 1996 ▶) *T*_min_ = 0.889, *T*_max_ = 0.952
10301 measured reflections
2338 independent reflections
1644 reflections with *I* > 2σ(*I*)
*R*_int_ = 0.037

#### Refinement

*R*[*F* ^2^ > 2σ(*F* ^2^)] = 0.043
*wR*(*F* ^2^) = 0.116
*S* = 1.03
2338 reflections
128 parameters
H-atom parameters constrained
Δρ_max_ = 0.33 e Å^−3^
Δρ_min_ = −0.47 e Å^−3^

### 

Data collection: *APEX2* (Bruker, 2007 ▶); cell refinement: *SAINT* (Bruker, 2007 ▶); data reduction: *SAINT*; program(s) used to solve structure: *SHELXS97* (Sheldrick, 2008 ▶); program(s) used to refine structure: *SHELXL97* (Sheldrick, 2008 ▶); molecular graphics: *PLATON* (Spek, 2003 ▶); software used to prepare material for publication: *WinGX* (Farrugia, 1999 ▶) and *PLATON*.

## Supplementary Material

Crystal structure: contains datablocks I, global. DOI: 10.1107/S1600536808035721/lh2721sup1.cif

Structure factors: contains datablocks I. DOI: 10.1107/S1600536808035721/lh2721Isup2.hkl

Additional supplementary materials:  crystallographic information; 3D view; checkCIF report

## Figures and Tables

**Table 1:** Hydrogen-bond geometry (Å, °)

*D*—H⋯*A*	*D*—H	H⋯*A*	*D*⋯*A*	*D*—H⋯*A*
N1—H1⋯O2^i^	0.86	2.20	3.054 (3)	174
O3—H8⋯O4^ii^	0.82	1.86	2.678 (2)	178

Symmetry codes: (i) 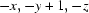; (ii) 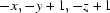.

**Fig. 1.**
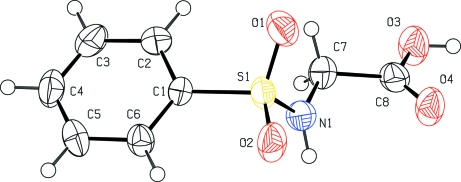
The molecular structure of the title compound showing the atom labelling scheme. Displacement ellipsoids are drawn at the 50% probability level.

**Fig. 2.**
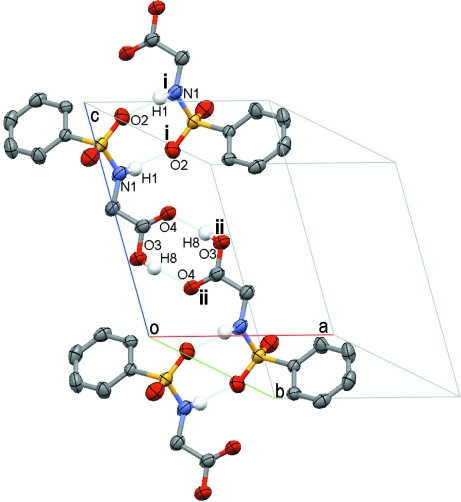
Part of the crystal structure, viewed approximately along the b axis, showing hydrogen bond interactions (dashed lines) along the [0 0 1] direction. H atoms not involved in hydrogen bonding have been omitted for clarity. Symmetry codes as given in Table 1.

## supplementary crystallographic information

### Comment

Sulfonamide is an important functionality found in many naturally occurring as well as synthetic compounds which possess numerous types of biological activities *e.g.*, anticonvulsant, antihypertensive, herbicidal and antimalarial (Soledade *et al.*, 2006; Esteve & Bidal, 2002; Xiao & Timberlake, 2000; Martinez *et al.*, 2000; Berredjem *et al.*, 2000; Lee & Lee, 2002) activities. In addition, these are found useful as anticancer (La Roche & Co, 1967) and antitubercular (Vaichulis, 1977) agents. These are also assumed as safe antibiotics during Pregnancy (Kayser *et al.*, 2004).

In the present paper, the structure of the title compound has been determined as a part of our ongoing research on the synthesis and biological evaluation of sulfur containing heterocyclic compounds (Zia-ur-Rehman *et al.*, 2005, 2006, 2007, 2008). In the molecule of (I) (Fig. 1), bond lengths and bond angles are almost similar to those in the related sulfonamide molecules (Gowda *et al.*, 2007*a*,*b*,*c*) and the bond lengths are within normal ranges (Allen *et al.*, 1987). In the crystal structure, each molecule is linked to an inersion related one through head-to-tail pairs of O—H···O intermolecular hydrogen bonds giving rise to dimeric motifs typical for carboxylic acids. Neighbouring dimers are further arranged into zigzag chains along *c* axis through head-to-tail pairs of N—H···O intermolecular interactions.

### Experimental

A mixture of *N*-benzenesulfonyl glycine methyl ester (1.0 g; 4.5 mmoles) and aqueous sodium hydroxide solution (10%; 10.0 ml) was refluxed for a peiod of one hour, cooled and acidified with 1 N hydrochloric acid. A white precipitate obtained was washed with water, dried and recrystallized from methanol to obtain colourless crystals suitable for X-ray studies; m.p. 435-436K.

### Refinement

All H atoms were placed in idealized positions, (C-H = 0.93-97 Å, O-H = 0.82Å, and N-H = 0.86Å), and included in the refinement in a riding-model approximation, with U_iso_(H) = 1.2U_eq_(C and N) and U_iso_(H) = 1.5U_eq_(O).

### Crystal data

**Table:**

C_8_H_9_NO_4_S	*F*_000_ = 448
*M**_r_* = 215.23	*D*_x_ = 1.535 Mg m^−^^3^
Monoclinic, *P*2_1_/*n*	Mo *K*α radiation λ = 0.71073 Å
Hall symbol: -P 2yn	Cell parameters from 2708 reflections
*a* = 8.5181 (3) Å	θ = 2.8–26.2º
*b* = 11.1302 (4) Å	µ = 0.34 mm^−^^1^
*c* = 10.6414 (4) Å	*T* = 296 (2) K
β = 112.600 (2)º	Cubes, colourless
*V* = 931.42 (6) Å^3^	0.36 × 0.16 × 0.15 mm
*Z* = 4	

### Data collection

**Table:**

Bruker APEXII CCD area-detector diffractometer	2338 independent reflections
Radiation source: fine-focus sealed tube	1644 reflections with *I* > 2σ(*I*)
Monochromator: graphite	*R*_int_ = 0.037
*T* = 296(2) K	θ_max_ = 28.4º
φ and ω scans	θ_min_ = 2.6º
Absorption correction: multi-scan(SADABS; Sheldrick, 1996)	*h* = −11→11
*T*_min_ = 0.889, *T*_max_ = 0.952	*k* = −14→13
10301 measured reflections	*l* = −14→14

### Refinement

**Table:**

Refinement on *F*^2^	Secondary atom site location: difference Fourier map
Least-squares matrix: full	Hydrogen site location: inferred from neighbouring sites
*R*[*F*^2^ > 2σ(*F*^2^)] = 0.043	H-atom parameters constrained
*wR*(*F*^2^) = 0.116	*w* = 1/[σ^2^(*F*_o_^2^) + (0.0498*P*)^2^ + 0.3567*P*] where *P* = (*F*_o_^2^ + 2*F*_c_^2^)/3
*S* = 1.03	(Δ/σ)_max_ = 0.001
2338 reflections	Δρ_max_ = 0.33 e Å^−^^3^
128 parameters	Δρ_min_ = −0.47 e Å^−^^3^
Primary atom site location: structure-invariant direct methods	Extinction correction: none

### Special details

**Table:**

Geometry. All e.s.d.'s (except the e.s.d. in the dihedral angle between two l.s. planes) are estimated using the full covariance matrix. The cell e.s.d.'s are taken into account individually in the estimation of e.s.d.'s in distances, angles and torsion angles; correlations between e.s.d.'s in cell parameters are only used when they are defined by crystal symmetry. An approximate (isotropic) treatment of cell e.s.d.'s is used for estimating e.s.d.'s involving l.s. planes.
Refinement. Refinement of *F*^2^ against ALL reflections. The weighted *R*-factor *wR* and goodness of fit *S* are based on *F*^2^, conventional *R*-factors *R* are based on *F*, with *F* set to zero for negative *F*^2^. The threshold expression of *F*^2^ > σ(*F*^2^) is used only for calculating *R*-factors(gt) *etc*. and is not relevant to the choice of reflections for refinement. *R*-factors based on *F*^2^ are statistically about twice as large as those based on *F*, and *R*- factors based on ALL data will be even larger.

### Fractional atomic coordinates and isotropic or equivalent isotropic displacement parameters (Å^2^)

**Table:**

	*x*	*y*	*z*	*U*_iso_*/*U*_eq_	
S1	0.16488 (6)	0.67470 (5)	0.09397 (5)	0.04003 (18)	
O1	0.1794 (2)	0.77973 (15)	0.17404 (17)	0.0549 (4)	
O2	0.01764 (18)	0.65761 (17)	−0.02937 (16)	0.0570 (5)	
O3	0.2307 (2)	0.51175 (18)	0.53583 (15)	0.0586 (5)	
H8	0.1631	0.5017	0.5728	0.088*	
O4	−0.0061 (2)	0.52365 (18)	0.34770 (15)	0.0573 (5)	
N1	0.1740 (3)	0.56105 (19)	0.18822 (18)	0.0518 (5)	
H1	0.1223	0.4965	0.1497	0.062*	
C1	0.3436 (2)	0.66945 (18)	0.04972 (19)	0.0319 (4)	
C2	0.5008 (3)	0.7030 (2)	0.1452 (2)	0.0474 (6)	
H2	0.5119	0.7290	0.2313	0.057*	
C3	0.6406 (3)	0.6970 (2)	0.1100 (3)	0.0575 (7)	
H3	0.7476	0.7174	0.1736	0.069*	
C4	0.6226 (3)	0.6612 (2)	−0.0184 (3)	0.0519 (6)	
H4	0.7171	0.6593	−0.0418	0.062*	
C5	0.4666 (3)	0.6282 (2)	−0.1123 (2)	0.0463 (5)	
H5	0.4558	0.6039	−0.1988	0.056*	
C6	0.3255 (2)	0.6309 (2)	−0.07854 (19)	0.0386 (5)	
H6	0.2197	0.6070	−0.1413	0.046*	
C7	0.2639 (3)	0.5602 (2)	0.3340 (2)	0.0456 (5)	
H7A	0.3143	0.6385	0.3640	0.055*	
H7B	0.3550	0.5015	0.3585	0.055*	
C8	0.1465 (3)	0.5299 (2)	0.4048 (2)	0.0429 (5)	

### Atomic displacement parameters (Å^2^)

**Table:**

	*U*^11^	*U*^22^	*U*^33^	*U*^12^	*U*^13^	*U*^23^
S1	0.0364 (3)	0.0515 (4)	0.0382 (3)	−0.0005 (2)	0.0210 (2)	−0.0011 (2)
O1	0.0665 (11)	0.0495 (10)	0.0606 (10)	0.0057 (8)	0.0376 (9)	−0.0035 (8)
O2	0.0327 (7)	0.0894 (14)	0.0493 (9)	−0.0016 (8)	0.0161 (7)	0.0003 (9)
O3	0.0539 (10)	0.0876 (14)	0.0368 (8)	−0.0090 (9)	0.0200 (7)	0.0061 (9)
O4	0.0496 (9)	0.0886 (14)	0.0364 (8)	−0.0138 (9)	0.0196 (7)	0.0028 (8)
N1	0.0702 (13)	0.0551 (13)	0.0395 (10)	−0.0230 (10)	0.0314 (9)	−0.0075 (9)
C1	0.0307 (9)	0.0338 (11)	0.0337 (9)	−0.0008 (8)	0.0153 (8)	0.0032 (8)
C2	0.0428 (11)	0.0554 (15)	0.0445 (12)	−0.0114 (10)	0.0171 (9)	−0.0138 (11)
C3	0.0350 (11)	0.0646 (17)	0.0718 (17)	−0.0140 (11)	0.0195 (11)	−0.0136 (14)
C4	0.0448 (12)	0.0513 (15)	0.0737 (16)	0.0012 (10)	0.0384 (12)	0.0036 (12)
C5	0.0544 (13)	0.0525 (14)	0.0430 (12)	0.0104 (11)	0.0308 (10)	0.0079 (10)
C6	0.0359 (10)	0.0494 (13)	0.0309 (10)	0.0026 (9)	0.0135 (8)	0.0044 (9)
C7	0.0501 (12)	0.0490 (14)	0.0441 (12)	−0.0058 (10)	0.0251 (10)	0.0018 (10)
C8	0.0550 (13)	0.0431 (13)	0.0344 (10)	−0.0059 (10)	0.0213 (10)	−0.0010 (9)

### Geometric parameters (Å, °)

**Table:**

S1—O1	1.4237 (17)	C2—H2	0.9300
S1—O2	1.4376 (16)	C3—C4	1.374 (4)
S1—N1	1.598 (2)	C3—H3	0.9300
S1—C1	1.7590 (19)	C4—C5	1.370 (3)
O3—C8	1.316 (2)	C4—H4	0.9300
O3—H8	0.8200	C5—C6	1.381 (3)
O4—C8	1.207 (3)	C5—H5	0.9300
N1—C7	1.443 (3)	C6—H6	0.9300
N1—H1	0.8600	C7—C8	1.502 (3)
C1—C6	1.382 (3)	C7—H7A	0.9700
C1—C2	1.385 (3)	C7—H7B	0.9700
C2—C3	1.380 (3)		
			
O1—S1—O2	119.98 (11)	C5—C4—C3	120.6 (2)
O1—S1—N1	107.60 (10)	C5—C4—H4	119.7
O2—S1—N1	106.46 (10)	C3—C4—H4	119.7
O1—S1—C1	107.60 (10)	C4—C5—C6	120.1 (2)
O2—S1—C1	107.00 (9)	C4—C5—H5	119.9
N1—S1—C1	107.66 (10)	C6—C5—H5	119.9
C8—O3—H8	109.5	C5—C6—C1	119.04 (19)
C7—N1—S1	123.95 (16)	C5—C6—H6	120.5
C7—N1—H1	118.0	C1—C6—H6	120.5
S1—N1—H1	118.0	N1—C7—C8	111.13 (18)
C6—C1—C2	121.18 (19)	N1—C7—H7A	109.4
C6—C1—S1	119.67 (15)	C8—C7—H7A	109.4
C2—C1—S1	119.14 (15)	N1—C7—H7B	109.4
C3—C2—C1	118.7 (2)	C8—C7—H7B	109.4
C3—C2—H2	120.7	H7A—C7—H7B	108.0
C1—C2—H2	120.7	O4—C8—O3	124.5 (2)
C4—C3—C2	120.3 (2)	O4—C8—C7	123.80 (19)
C4—C3—H3	119.8	O3—C8—C7	111.68 (19)
C2—C3—H3	119.8		
			
O1—S1—N1—C7	−28.4 (2)	S1—C1—C2—C3	179.16 (19)
O2—S1—N1—C7	−158.27 (18)	C1—C2—C3—C4	1.5 (4)
C1—S1—N1—C7	87.27 (19)	C2—C3—C4—C5	−1.6 (4)
O1—S1—C1—C6	−141.46 (18)	C3—C4—C5—C6	0.1 (4)
O2—S1—C1—C6	−11.3 (2)	C4—C5—C6—C1	1.3 (4)
N1—S1—C1—C6	102.82 (18)	C2—C1—C6—C5	−1.3 (3)
O1—S1—C1—C2	39.3 (2)	S1—C1—C6—C5	179.46 (17)
O2—S1—C1—C2	169.46 (18)	S1—N1—C7—C8	121.9 (2)
N1—S1—C1—C2	−76.44 (19)	N1—C7—C8—O4	−9.4 (3)
C6—C1—C2—C3	−0.1 (3)	N1—C7—C8—O3	170.8 (2)

### Hydrogen-bond geometry (Å, °)

**Table:**

*D*—H···*A*	*D*—H	H···*A*	*D*···*A*	*D*—H···*A*
N1—H1···O2^i^	0.86	2.20	3.054 (3)	174
O3—H8···O4^ii^	0.82	1.86	2.678 (2)	178

Symmetry codes: (i) −*x*, −*y*+1, −*z*; (ii) −*x*, −*y*+1, −*z*+1.
